# Editorial: Advances in the involvement of brain cellular subpopulations and pathways in distress and stress-related disorders

**DOI:** 10.3389/fnins.2023.1264545

**Published:** 2023-08-29

**Authors:** Gessynger Morais-Silva, Cristiane Aparecida Favoretto, Marco Pagliusi, Tara A. LeGates

**Affiliations:** ^1^São Paulo State University (UNESP), School of Pharmaceutical Sciences, Department of Drugs and Medicines, Laboratory of Pharmacology, Araraquara, São Paulo, Brazil; ^2^Universidade Federal de São Paulo (UNIFESP), Department of Pharmacology, Molecular and Behavioral Neuroscience Laboratory, São Paulo, São Paulo, Brazil; ^3^University of São Paulo (USP), Ribeirao Preto Medical School, Department of Pharmacology, Ribeirao Preto, São Paulo, Brazil; ^4^Department of Biological Sciences, University of Maryland, Baltimore County (UMBC), Baltimore, MD, United States; ^5^Department of Physiology, University of Maryland School of Medicine, Baltimore, MD, United States

**Keywords:** stress, neurons, glia, neuronal subpopulations, cell phenotypes

Chronic stress exposure represents a prominent risk factor for the development of neuropsychiatric disorders, including depression, anxiety, substance use disorders, and post-traumatic stress disorder (PTSD), significatively affecting the well-being and health of millions of individuals worldwide (McEwen and Akil, 2020). Heterogeneity in stress outcomes have complicated our understanding of the neurobiological basis of stress effects on the brain and related psychiatric disorders. In this Research Topic, we aimed to bring together original research and reviews addressing the role of cell subtypes, neural pathways, and brain regions in stress- and distress-related effects on physiology and behavior.

Abnormal persistence and reactivation of stressful or fearful experiences are a key characteristic of PTSD. The work by Inoue et al. evaluated whether disrupting reconsolidation of a fear memory could weaken the original memory. To examine the neurobiological factors involved, the authors ablated hippocampal neurogenesis and downregulated glutamatergic NMDA receptor signaling via D-serine—a potent NMDA receptor co-agonist—blockade and tested retrieval 1, 7, 14, and 28 days after fear conditioning training. In a series of experiments, they show that brief retrievals induce reconsolidation, and blockade of D-serine signaling and neurogenesis blocks this reconsolidation and attenuates remote fear memory (at day 28). Together, this work suggests D-serine signaling and hippocampal neurogenesis have a key role in promoting contextual fear memory by promoting reconsolidation.

Epidemiological studies suggest a relationship among age-related hearing loss (AHRL), tinnitus, and psychiatric diseases related to stress exposure (Loughrey et al., 2018; Du et al., 2023). Looking into this subject, the review by Ruan et al. brought insights into the phenotypic heterogeneity of AHRL and tinnitus, and the contribution of chronic non-audiogenic and chronic audiogenic stressors to such heterogeneity. They elegantly reviewed literature regarding the chronic effects of stress exposure on limbic brain structures and on those systems closely related to auditory processing, and how such effects could lead to different frailty phenotypes and aggravate AHRL and/or tinnitus.

In “*Determination of steady-state transcriptome modifications associated with repeated homotypic stress in the rat rostral posterior hypothalamic region*”, Campeau et al. investigated the transcriptional regulations related to the habituation to homotypic stress exposure in the hypothalamus. Male Sprague-Dawley rats exposed to 7 days of loud noise showed a reduction in noise-induced increases in plasmatic corticosterone compared to rats exposed to 1 or 3 days of loud noise exposure. This neuroendocrine habituation was accompanied by a large number of genes that were differentially expressed in the rostral posterior hypothalamic region 24 h after the last stress exposure, a time point when such changes should be stable. Gene ontology analyses revealed that transcripts associated with neuron differentiation, neural membrane potential, pre- and post-synaptic elements, chemical synaptic transmission, vesicles, axon guidance and projection, glutamatergic and GABAergic synapses were differentially regulated, and authors argued that such alterations could contribute to stress habituation.

It is known that the dysregulation of circadian clocks has been implicated in several stress-related diseases, mainly mood and anxiety disorders. Based on this, the review by Francis and Porcu gathers the literature regarding cell-type specific influence on mood-related behaviors and the circadian system. Despite the small number of works within the scope of this review, the authors highlight the suprachiasmatic nucleus, ventral tegmental area, nucleus accumbens, habenula, thalamus, dorsal raphe nucleus, hippocampus, and amygdala, discussing the impairment of cellular communication and neuronal circuitry related to stress and circadian-driven mood disruption.

da Costa et al. aimed to investigate the impact of chronic social defeat stress on depressive-, anxiety-, and cognitive-like behaviors, and the activation of glutamatergic neurons in the bed nucleus of stria terminalis (BNST), amygdaloid complex, and hippocampus of adult male mice 48 h after termination of repeated stress exposure. Results showed that chronic social defeat induced social avoidance, increased anxiety-like behaviors, and impaired short-term memory. Although social defeat did not induce a clear depressive-like phenotype, it led to increased deterioration of coat state and weight gain. Moreover, social defeat increased ΔFosB expression in glutamatergic neurons in BNST and amygdala, increased ΔFosB immunoreactivity in the ventral hippocampus, and reduced ΔFosB labeling in the dorsal hippocampus. The authors discuss the potential relationships between the behavioral outcomes and the immunoreactivity patterns observed, suggesting that differential activity may underlie behavior.

Favoretto et al. expand on this idea of cell-type differences as they review how different cell types in the brain contribute to stress susceptibility. Beginning with non-neuronal cells, they discuss how astrocytes and microglia respond to stress, and how functional changes in these two cell populations are related to stress outcomes. They then explore relationships between stress-induced changes in neuron function and behavioral outcomes focusing on a specific collection of neuronal subtypes. This discussion also highlights key studies that have experimentally manipulated these cell types to establish causal relationships between neuronal activity and behavior. This focused, yet thorough, review of the literature provides important insight into the cellular and molecular diversity that underlies responses to stress, which is an important consideration for understanding the pathophysiology of stress-related disorders and discoveries of next-generation treatments.

The work presented in this Research Topic highlights the multifaceted effects of stress from molecules to behavior. Unraveling the complexities of stress and its effects on the brain will be important for understanding the neurobiological basis of stress responses with key insight into the pathophysiology underlying stress-based psychiatric disorders. Advances in understanding the involvement of precise neural substrates on stress-related outcomes may unravel specific cellular and molecular targets for the development of next-level therapies for neuropsychiatric disorders. The editors hope you enjoy this Research Topic and that it will be useful and insightful to your research.

## Author contributions

GM-S: Writing—original draft, Writing—review and editing. CF: Writing—original draft, Writing—review and editing. MP: Writing—original draft, Writing—review and editing. TL: Writing—original draft, Writing—review and editing.
